# The Evolving Role of Pharmacists in Transgender Health Care

**DOI:** 10.1089/trgh.2018.0038

**Published:** 2019-04-11

**Authors:** Jan S. Redfern, Michael W. Jann

**Affiliations:** ^1^ Department of Pharmacotherapy, University of North Texas System College of Pharmacy, Fort Worth, Texas.; ^2^ Redfern Strategic Medical Communications, Inc., Springtown, Texas.

**Keywords:** cultural competency, health care, pharmacist, pharmacy education, transgender

## Abstract

Pharmacists are increasingly part of a multifaceted team providing health care to members of the often marginalized transgender (TG) community. Some pharmacists, however, may feel unprepared to care for and interact with TG individuals. By providing comprehensive, respectful, and gender-affirming support, improving physical pharmacy environments with policies and procedures, pharmacists can be trustworthy providers for TG patients. This review focuses primarily on the health issues of TG persons and the pharmacist's role in promoting health, identifying barriers to health care, and providing health care resources for TG persons. The evolution of psychiatric diagnostic criteria, access to health care, and inclusion of TG, lesbian, gay, and bisexual topics in the educational curriculum are presented. Cultural competency and diversity training that addresses gender identity and sexual orientation issues should be important interdisciplinary and interprofessional activities for all health care professional education programs. Pharmacists play a key role in the health care needs of TG persons that include appropriate laboratory monitoring, complex pharmacotherapeutic challenges, and providing unbiased gender-affirming interactions. The pharmacy's physical environment, staff training, and policies and procedures can offer unique services to TG persons.

## Introduction

The role of the pharmacist has changed considerably over the past decade, in part, as a result of the introduction of automated prescription filling systems and greater responsibilities of pharmacy technicians.^1–4^ Pharmacists now find they have greater opportunities to become a more essential and visible component of individualized patient care. Although the exact number of pharmacists involved with transgender (TG) health care is unknown, they are included as part of a multifaceted team delivering health care to the TG and lesbian, gay, and bisexual (LGB) community (e.g., as part of the Fenway Health's multidisciplinary health care model).^5^ Indeed, pharmacists can be approachable, reliable providers for TG patients who may feel safer sharing their concerns or issues with a pharmacist rather than a physician.^6^ Thus, pharmacists play an important role as a partner with primary care providers. Both clinical pharmacists and psychiatric pharmacists (with behavioral and pharmacologic expertise) have been introduced as crucial part of an interprofessional team to provide health care to TG veterans.^7^

TG patients experience marked bias in the health care system, and this leads to numerous health care disparities as well as impediments to care.^8,9^ Pharmacy practice is not “immune” to the discriminatory behavior toward TG persons.^10^ Yet, pharmacists can help promote the health of their TG patients by examining and updating their patient interactions, pharmacy environments, policies, and staff training. Fostering these activities will encourage TG patients to return to the pharmacy for their continued care and receive important health care advice when they experience welcoming, positive, and unbiased pharmacy staff who are educated about the TG community. The TG and LGB community and resource centers typically are aware of and refer via “word of mouth” health care professionals and various practices that provide welcoming patient care services for TG and LGB individuals. Pharmacy practice has also evolved creating community pharmacy practice settings and residency programs specializing in human immunodeficiency virus (HIV), hepatitis C, hormonal therapies, and other therapeutic areas.^11,12^

**FIG. 1.**
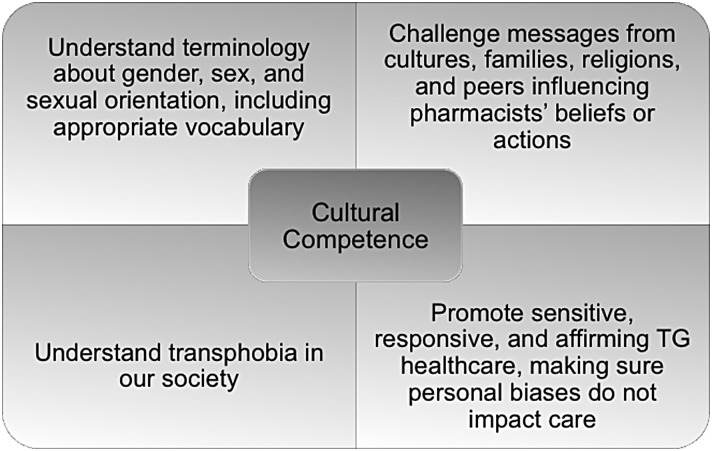
Cultural competency of pharmacists in TG health care involves at least four major facets. TG, transgender.

Health care professionals in general, including pharmacists, may feel uncomfortable in caring for and interacting with TG individuals.^8,9^ These situations can be compounded by the absence of TG and LGB topics in the curriculum of many pharmacy programs.^13^ Surprisingly, unlike the medical profession, the American Pharmacists' Association and other pharmacy organizations have not published any policy statements on the role of pharmacists in caring for TG patients.^14–16^ Meeting the health care needs of TG people, however, will require pharmacists, like all health care practitioners, to embrace cultural competency (Fig. 1) in their practices and augment their awareness and receptivity to the growing TG population. Cultural competency and awareness of health disparities are key elements in the pharmacy school curriculum. Although the Accreditation Council for Pharmacy Education includes cultural competency as part of its accreditation standards and guidelines, these statements are vague and nonspecific regarding the LGBT population.^17^ The provision of culturally competent care for pharmacists should begin in the student setting when LGBT topics are included in the curriculum.

This current article provides a succinct overview of TG culture, prevalence, and etiology for the interested pharmacist and serves as a primer on the health care issues of TG persons and the pharmacist's role in providing high-quality and culturally competent care.

## Methodology

An *a priori* structured search of the PubMed database was initially utilized to develop a comprehensive review of the health care of TG persons in the pharmacy setting. Original research, review articles, and editorials (1995–2018) in the English language were identified using a search strategy that combined the terms gender identity disorder, gender dysphoria, TG, or transsexual with the terms pharmacy, pharmacist, or pharmacy education. A total of 63 unique articles were initially retrieved, of which 15 specifically addressed issues directly relevant to the role of pharmacists in TG health care.^13,18–31^ Additional references (one journal article^32^ and five web-based articles^6,10,33–35^) were identified by searching electronic databases (Google Scholar, Google, Science Direct, and Cochrane Database of Systematic reviews) and by manually searching the reference lists of identified articles. Other secondary searches (for various subsections) were also performed utilizing the terms transgender or gender dysphoria or gender identity disorder and epidemiology, etiology, disease management, diagnosis, cultural competency, pharmacotherapy, health care, human immunodeficiency virus, sexually transmitted disease, and antiretroviral therapy.

## TG Covers a Wide Spectrum of Individuals

The term TG encompasses a wide spectrum of individuals whose gender identity, gender expression, or behavior do not conform to that typically associated with the sex assigned to them at birth (TG women or trans women: female gender identity and a male birth-assigned sex, and TG men or trans men: male gender identity and a female birth-assigned sex).^9^ Using data from CDC's Behavioral Risk Factor Surveillance System, the Williams Institute in Los Angeles, California, estimates the prevalence of TG people in the US population to be ∼0.6% or 1.4 million adults, roughly the population of Hawaii.^36^

Some, but by no means all, TG people experience marked distress with their sex-assigned at birth, and this can trigger their decision to transition—that is, to change their physical, social, and legal characteristics to the gender opposite that of their sex assigned at birth (biologic sex).^8^ Transition is a complex, individualized process and may be accompanied by treatment with feminizing or masculinizing hormones (hence a point of contact of TG individuals with pharmacists) to help transform their physical appearance to match their gender identity. It is important to note, however, that not all TG people undergo surgeries or take hormones. According to the 2015 US Transgender Survey, only 12% of TG women and 3% of TG men undergo vaginoplasty or phalloplasty, respectively.^37^ The most frequent procedure in TG men is chest surgery (“top surgery” in common parlance), followed by hysterectomy (14%). In TG women, the most frequent procedure is hair removal (47%) followed by voice therapy (14%). The overall therapeutic goal for TG individuals is to obtain lasting personal comfort and self-acceptance in terms of body and gender role through affirmative psychotherapy and medical and/or surgical therapies.

Approximately half of TG patients receive cross-sex hormone therapy, although a majority of TG persons (78%) desire hormonal pharmacotherapy.^37^ Only small fraction of TG persons (11%) have been able to change all of their legal identification (e.g., driver's license, etc.) to their preferred name and gender while the majority (68%) had not changed their identity at all.^37^ This has important implications for health care professionals, including pharmacists, in terms of patient interactions and processing medication insurance claims.^38^ The appearance of TG individuals often does not match their legal (e.g., federal, state, or county) identification documents. Pharmacists are likely to encounter TG individuals at various stages of transition with unexpected physical combinations (women with a prostate or men with a cervix or even pregnancy).

## Evolution of the Diagnostic Criteria in Psychiatry Regarding TG Persons

Biologic, environmental, and cultural factors are all believed to play a role in the evolution of gender identity (to be distinguished from biological or genotypic sex).^39^ Gender nonconformity, according to the American Psychological Association, is “not in itself a mental disorder.”^40^ Rather, it may be perceived as nothing more than variation of human development. TG *per se* is not a clinical term or a diagnosis and does not appear in DSM-5 or any other diagnostic manual. Instead, the clinical diagnosis is gender dysphoria. Gender dysphoria is characterized as a marked difference between gender identity and birth sex and a desire to be treated as the other gender and change sex characteristics.^40^ The main component of gender dysphoria is the clinically significant distress it causes and the marked impairment of social, occupational, or other important areas of functioning. It is important to note, however, that being TG does not suggest a particular sexual orientation. Gender identity and sexual orientation are disparate factors contributing to the overall sexual construct of an individual.^9^

Experts in the field changed the diagnosis from gender identity disorder in DSM-IV to gender dysphoria in DSM-5 with the aim of depathologizing the condition and focusing more on the distress experienced by individuals (while preserving a diagnosis acceptable for third party insurance coverage).^41–43^ In recent years, a trend has emerged, in which gender-affirming models have focused on identity issues rather than a pathological disorder.^44^ The trend of depathologization is also mirrored in the proposed changes for ICD-11. The diagnostic classification may be changed from the rather archaic ICD-10 designation of transsexualism (F64.0) located in the mental health and behavioral disorders section to gender incongruence in the conditions related to sexual health in ICD-11.^45,46^

The terms TG, transsexual, gender dysphoria, or gender incongruence will likely follow the same depathologizing path as homosexuality, a term that was first changed to sexual orientation disturbance in DSM-III in 1973 and then completely removed as a psychopathology in DSM-III-R in 1987.^47^ It can be contended that the mental health issues experienced by TG individuals (e.g., depression, anxiety, substance abuse, suicidal ideation) arise more from the hostile social forces repressing gender nonconformity and creating psychosocial stress (minority stress model) rather than intrinsic to TG condition itself.^48^ A comparative study in Colorado noted that the incidence of depression and anxiety was significantly higher in TG individuals compared with the general population (43% vs. 6.8% and 52% vs. 15%, respectively). The prevalence of suicidal ideations and suicide attempts in the previous year were also significantly greater in TG individuals than in the general population (36% vs. 4% and 10% vs. 0.8%, respectively).^49^

Not all professional organizations support TG persons considering or undergoing the transition process. For example, the American College of Pediatricians issued a position statement urging “health care professionals, educators, and legislators to reject all policies that condition children to accept as normal a life of chemical and surgical impersonation of the opposite sex. Facts—not ideology—determine reality.”^50^ Further, the Department of Health and Human Services (DHHS) is currently directing an effort to formulate a legal definition of sex under Title IX. A recent DHHS memo leaked to The New York Times stressed the need for government agencies to adopt an unequivocal and consistent definition of gender: that is, “sex as either male or female, unchangeable, and determined by the genitals that a person is born with.”^51^ It remains to be seen what impact this will have on TG individuals in terms of civil rights protection and health care coverage under the Affordable Care Act.

While there is no definitive explanation for gender dysphoria, a number of potential underlying causes have been proposed involving prenatal/postnatal sex hormone effects, infant and adolescent experiences, genetic influences, and neuroanatomical differences (e.g., in the bed nucleus of stria terminalis, corpus callosum, and uncinate nucleus).^52–56^ Phenotypes for TG women and men in terms of neural structure and connectivity have been put forward based on functional magnetic resonance imaging studies.^57^

## Access to TG Health Care

Many TG persons encounter difficulty in obtaining culturally competent health care, with individuals in rural versus urban settings facing the greatest challenge, and often experience providers lacking knowledge of TG health issues (Table 1).^58,59^ A recent survey of internal medicine and family medicine physicians and residents who practiced in a Midwest health system found that only 50% of respondents were willing to continue administering hormone therapy to TG patients.^60^ Another survey of internal medicine residents at a large urban academic center found that only 9% of respondents felt confident prescribing hormone therapy.^61^

**Table 1.** Principal Barriers to Health Care for Transgender Individuals

Lack of providers with expertise in TG medicine
Financial barriers
Lack of insurance
Lack of income
Discrimination
Lack of cultural competence by health care providers
Health systems barriers
Inappropriate electronic records
Forms
Laboratory references
Clinic facilities
Socioeconomic barriers
Transportation
Housing
Mental health

TG, transgender.

*Source:* Safer et al.^58^

TG individuals fear being disrespected, misgendered, inappropriately questioned, and, if not refused treatment outright, treated grudgingly in demeaning manner.^9,62^ Not surprisingly, therefore, TG people avoid or delay health visits such that preventable illnesses are not addressed, complications worsen, and costs increase.^8^ According to an analysis of 3486 TG patients who were part of the National Transgender Discrimination Survey, almost 31% of TG individuals said that they had delayed or failed to pursue needed health care because of discrimination.^63^ The delay in health care due to intolerance by society has been also reported with LGB persons.^64^ The delay in health care for TG individuals can increase the burden of disabilities given the existence of other multiple chronic medical conditions.^65^

Access to health care may be further impeded in light of the DHHS plans to form a Conscience and Religious Freedom Division within its Office for Civil Rights.^66^ It remains to be seen whether this new Division would make it easier for health care providers and hospitals to deny treatment to TG patients if it conflicts with their individual beliefs.

Based on results of physician interviews in Ontario, there are several reasons that the health care providers may feel uncomfortable treating TG patients.^67^ Some providers consider the condition of being TG as a reversible psychological issue best treated with psychiatric intervention, a finding that highlights their knowledge/training deficits. In addition, personal convictions, ethical considerations pertaining to transition-related medical care, unrealistic patient expectations, or perceived legal liability over potential regret of TG persons after undergoing irreversible hormonal or surgical treatments also represent additional barriers to TG health care. However, regret following gender affirmation procedures among TG individuals is typically very low (0–3.8%).^68–70^ The health care system as a whole also offers challenges in terms of recognizing and accommodating TG persons into a system segregated into a binary-sex designation and the difficulty in identifying TG-friendly physicians for specialist referrals. Nevertheless, over the past decade, attitudes of health care providers toward TG persons appear to be trending in a positive direction. Education of health care providers in TG-related issues is a key component toward favorably impacting the dynamics of the provider/TG patient interaction.

## Inclusion of LGBT Topics into the Pharmacy Curriculum

A survey of 130 pharmacy school administrators identified accessible gender-neutral/single-occupancy restrooms and availability of LGBT trainings, scholarships, and events as key areas for improvement.^26^ Most pharmacy programs lack inclusion of many LGBT health topics in their curriculum, campus-wide events, and student organizations. The survey also reported that a mere one-fifth of the programs have inclusive materials for the faculty, staff, and students about sexual orientation and gender identity.^26^ Each group within the LBGT population has its own unique challenges in health care. Regarding TG persons, their specific and unique health care issues should be addressed in any pharmacy curriculum.^33^ The impact of a diversity course that was offered to first-year pharmacy students and included a TG panel discussion of both trans men and trans women is particularly noteworthy.^22^ The students reported that the panel discussion was an eye-opening experience and relevant to their pharmacy careers.^22^ Commentaries have been published for pharmacy programs to include and pharmacists to be culturally competent and educated in LGBT health with opportunities for research.^13,24,28^

Recently, an elective two-credit course on LGBT health care was implemented at UNT System School of Pharmacy focusing on health promotion, identifying barriers to health care, and health care resources for LGBT persons (Table 2).^32^ Several guest speakers from the LGBT community participated in the course. The comparison of the pre- vs. postcourse seven learning objectives (e.g., summarizing health care resources for LGBT persons) reported significant improvement (*p*<0.001) in the students. The course included four specific medical didactic classes in the health care of TG persons, a panel discussion with TG persons in transition, and an objective structured clinical examination (OSCE) activity. The student OSCE activity was a 10-min counseling session with a TG patient and their treatment with a hormone prescription.^71^ At the course's conclusion, the student evaluations were very positive with student comments stating how the course enhanced their skills and knowledge relating to the LGBT community. Similar findings were described by Ostroff et al. who reported that students who attended a lecture on cultural competency and pharmacotherapy relating to TG individuals performed better than those who did not with respect to knowledge-based assessment and confidence in interacting with TG individuals.^20^ Cultural competency and diversity should be an interdisciplinary and interprofessional activity for all health care professional education programs.^31,72^

**Table 2.** Summary of Material Covered in an Elective LGBT Course for Pharmacists

Health care professionals' awareness and knowledge of health risk, disparities, and potential resiliency of people who are
LGB
TG or gender nonconforming
Individuals born with DSD
Social, economic, and ethical issues related to the LGBT persons
Roles of health care professionals and the various services and venues available in the care of LGBT persons
Provision of high-quality, patient-centered care to persons who are LGBT, gender nonconforming, and/or born with DSD
Mental and physical health issues and pharmacotherapeutic strategies in LGBT persons
Practice guidelines, evidence-based medicine, and population-based treatment plans to the relevant disease(s)
Patient-specific regimens to treat the relevant disease(s) utilizing patient-specific parameters (including the complexities of using multiple drug classes and/or the presence of comorbid conditions or organ dysfunction)
Advocacy for the health of LGBT persons

DSD, differences of sex development; LGB, lesbian, gay, and bisexual.

Pharmacy school faculty advisors, educators, and administrators can play a critical role in helping TG students, particularly those actively transitioning, to overcome barriers and provide a safe and nondiscriminatory learning environment.^21,30^ Graduating LGBT student pharmacists who are interested in postdoctoral residency training will need to evaluate both personal and professional situations and determine the level of disclosure while developing strategies for disclosure during the application process.^19^

## The Pharmacist's Role in the Health Care Needs of TG Individuals

TG persons not only have the same basic health needs in terms of screening, prevention, and treatment as their non-TG counterparts but also have clinical issues specifically related to or associated with gender incongruence (Table 3). Many of these issues are relevant to pharmacists and may directly or indirectly impact the medical care of TG patients. These include, for example, depression, alcohol or substance abuse, anxiety, suicidal tendencies, increased susceptibility for sexually transmitted diseases (STDs; especially HIV) due to high-risk behaviors, and other medical conditions resulting from the side effects of cross-sex hormone therapy.

**Table 3.** Transgender Health-Related Issues of Potential Relevance to Pharmacists

Hormones
Testosterone, estrogen, progestin (illicit or prescribed)
Testosterone-blocking agents (cyproterone acetate, spironolactone)
Gonadotropin-releasing hormone analogs to delay puberty in TG children
Cross-sex hormone side effects
Cardiovascular and cerebrovascular disease, thromboembolic disease
Liver damage, cholelithiasis
Type 2 diabetes
Blood lipids, lipoproteins, pancreatitis
Thromboembolism
Hyperprolactinemia
Laboratory test abnormalities
Drug–drug interactions
Cancer
Reproductive organ cancers such as prostate cancer, cervical or ovarian cancer, and breast cancer
Mental health issues
Depression, anxiety
Suicidal ideation, gestures and attempts
Self-harm (especially in teenagers)
Decreased expectations related to relationship, educational and career success
STDs
HIV/AIDS and other STDs, PrEP
Hepatitis
Substance and alcohol abuse
Alcohol abuse and dependence
Abuse of “party drugs” such as cocaine, crystal meth, ecstasy, ketamine, bath salts, synthetic marijuana (K-2)
Tobacco use
Cigarettes, other tobacco products, e-cigarettes
Heart disease
Hypertension
Lipid dyslipidemia
Diabetes

HIV, human immunodeficiency virus; PrEP, preexposure prophylaxis; STDs, sexually transmitted diseases.

Ten key principles have been recommended to assist providers to create a warm environment and provide quality care of TG persons.^73^ The National Institutes of Health Sexual and Gender Minority Research Coordinating Committee recognize the importance of the health and well-being of LGBT individuals and have announced a 5-year (2016–2020) strategic plan to advance research on basic, clinical, and behavioral and social sciences.^74^ In addition, the Health People 2020 initiative of the US Department of Health of Health and Human Services added LGBT individuals specifically to their overarching goal of eliminating health disparities, improving quality of life, and promoting good health.^75^

Pharmacotherapy of TG persons is complex and involves a large number of medications with most of the focus on hormone treatment during and after transition.^18^ Hormonal products are not FDA approved for TG persons and their use is considered “off-label.” The time for maximal effects from hormone treatments can take up to 2–3 years with antiandrogen and estrogen therapy and 4–5 years with testosterone therapy. However, these time periods are estimates based on patients undergoing uninterrupted treatment. Patients should be aware that overdosing with testosterone, aside from increasing the risk of adverse events, may actually produce feminizing effects since testosterone at high doses is converted to estrogen via aromatase. As many TG persons are without health insurance,^76^ these therapies are often episodic and, thus, the desired physical changes take longer to achieve adding to the complexity of treatment. Pharmacists are ideally positioned to assist TG persons with medication access programs, especially since testosterone is categorized as a Schedule III controlled substance. A wide variety of testosterone products are available. Pharmacists can assist TG persons in education of the different injection products such as the safety methods with single use ampules, distinguishing between single use versus multidose vials, or when products are changed. Injection site selection, technique, rotation, and safety for long-term treatment can also be included during the pharmacist's consultation with TG persons. Providers generally rely on published treatment guidelines but frequently need to modify treatment regimens based upon cost factors and changing protocols over time. For example, the Endocrine Society updated its clinical practice guidelines for TG persons.^15^

As previously mentioned, TG persons typically also have multiple chronic medical conditions (e.g., diabetes, asthma, arthritis, coronary heart disease, HIV, hepatitis), mental health issues, smoking, episodic binge drinking (defined as >7 drinks/week for women and >14 drinks for men), and lack of vaccinations, and together they present complex and challenging treatment issues for the pharmacists.^65^ Many TG persons have limited access to health care as <60% of TG persons reported employment and <28% reported incomes greater than $50,000 per year.^65^ Pharmacists can benefit the treatment team and the TG person by (1) increasing access to medications through contact with various pharmaceutical industry programs, federal, state, and local agencies, (2) by ensuring the most cost-effective medications are dispensed, (3) discussing the most appropriate dosage regimens and formulations, (4) discussing the risks and benefits of cross-sex hormone therapy, (5) carefully monitoring of patients for efficacy and safety, and (6) suggesting risk-reduction strategies (smoking cessation and weight loss).^25,29^ Finally, updating treatment protocols as new data emerges is a unique role for pharmacists.^77^ Other indirect considerations for pharmacists in the health care aspects for TG individuals are positive reinforcement of therapies, assisting in social transitions, and possible medication effects (e.g., anticholinergic drugs and dry mouth) on voice and speech pattern training.

## Laboratory Measurements in TG Individuals

Pharmacists, particularly those involved in TG health care in hospital settings as part of an integrated team, should be familiar with clinical guidelines recommending laboratory monitoring of TG patients on cross-sex hormone therapy. They should also have access to laboratory findings to explore any discrepancies in values. However, this raises a very important question: “What gender-specific reference intervals are ‘normal' for each patient?” This issue was addressed in a small study comparing laboratory results from 55 TG females on hormone therapy with 20 male and 20 female cisgender subjects (i.e., individuals whose gender identity or expressions align with assigned sex at birth).^78^ The study found that hemoglobin, hematocrit, and low-density lipoprotein cholesterol laboratory values resembled those found in cisgender females (*p*<0.005) while alkaline phosphatase, potassium ion, and creatinine resembled those values found in cisgender males (*p*<0.05). On the contrary, triglyceride levels were higher (*p*<0.005) than either cisgender male or female groups. Discrepancies in creatinine clearance are particularly important since renal function often impacts dosing protocols of many agents. These findings have important implications in that almost half of diagnostic errors occur at the level of the laboratory testing (e.g., failure to order, report, and follow-up laboratory results).^79^ Due to limitations in laboratory reference values, it is imperative that reference ranges be established for TG male or TG females to avoid unnecessary evaluations, misdiagnoses, or unwarranted therapy.

## TG Individuals and STDs

**FIG. 2.**
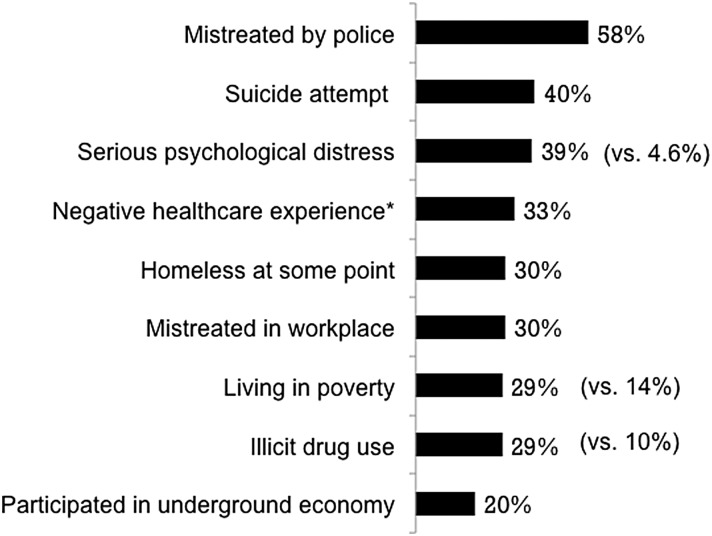
TG individuals face numerous challenges in many aspects of their daily lives. Data extracted from the 2015 US Transgender Survey of 27,715 respondents.^37^ *Verbally harassed or refused treatment because of their gender identity. Corresponding data for the general population are given in parentheses.

TG individuals face many challenges in their everyday lives, including mistreatment by police, homelessness, poverty, and negative health care experiences (Fig. 2).^37^ Not surprisingly, many TG people experience serious psychological distress that can lead to suicidal ideations and suicide attempts in some cases. Some TG women live at the margins of society and engage in drug use and sex work for their basic survival. These activities place TG women at a high risk for violence and STDs.

**FIG. 3.**
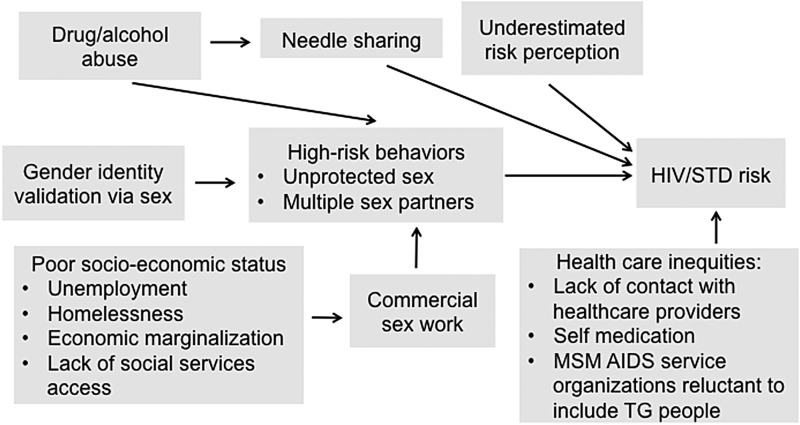
Factors contributing to the increased risk of HIV/STD risk in TG people. HIV, human immunodeficiency virus; STD, sexually transmitted disease. Reproduced with permission from Redfern et al.^8^

The burgeoning prevalence of HIV/AIDS in TG individuals is of special concern as TG women carry the heaviest burden compared with other LGB groups and TG men. The prevalence of HIV in TG women globally averages 20% but laboratory-confirmed HIV prevalence in TG communities in San Francisco and New York may be as high as 35% and 40%, respectively.^80,81^ The odds of HIV infection in TG women is a staggering 49-fold greater compared with the general adult population of reproductive age.^80^ Reback and Fletcher examined HIV prevalence, substance abuse, and sexual risk behaviors among TG women encountered on the streets and at high-risk venues in Los Angeles.^82^ The study showed that TG women (*N*=2136) had many risk cofactors for HIV infection and transmission, including high rates of recent alcohol (57.7%), marijuana (25.6%), and methamphetamine (21.5%) use, lifetime injection drug or illegal hormone use (66.3%), and recent engagement in sex work (73.3%). A variety of factors (Fig. 3), including high-risk sexual behaviors, underestimated perception of risk behavior, mental health issues, and health care inequities contribute to the increased risk of STD/HIV infection among TG women.^8^ These statistics are compounded by the fact that TG women are less likely to access and utilize HIV services.^83^

The WHO recognizes this enormous burden, noting that the “high vulnerability and specific health needs of TG people necessitates a distinct and independent status in global HIV response.”^84^ Clinics providing hormones to TG represent a valuable entry point to obtain HIV care since TG individuals often prioritize hormones over HIV care.^84^ Indeed, there are anecdotal cases of TG individuals deliberately exposing themselves to HIV to become eligible to attend HIV clinics and thereby obtain hormones.^8^ Bolstering HIV prevention, treatment, and care targeted to TG people is certainly a key goal for the future.^85,86^ HIV risk behaviors and risk determinants may be attenuated through combining TG-specific medical and psychosocial services with targeted HIV care.^87^ The Human Rights Campaign Foundation has provided useful guidelines on the involvement of pharmacists in HIV care (Table 4), which includes, among other things, offering on-site rapid HIV testing, counseling services, identifying TG patients who may benefit from preexposure prophylaxis (PrEP), and linking them to prescribers.^34^ Appropriate referrals by pharmacists can assist TG persons for HIV testing and guidance for postexposure prophylaxis (PEP) or PrEP considerations (PEPline 1-888-448-4911 or PrEPline 1-885-448-7737). A recent study of men who have sex with men suggests pharmacies should consider reinforcing information on over-the-counter HIV tests, which are preferred to pharmacy-based testing, particularly in rural areas, and providing opportunities for consultation and referral for care.^88^

**Table 4.** Pharmacists' Role in HIV Prevention and Treatment in Transgender Individuals

Refer patients to community HIV testing sites
Offer on-site rapid HIV testing and counseling services
Make referrals for confirmatory testing and linkage to care
Help to identify patients who may benefit from PrEP and linking them to prescribers
Assist patients in payment assistance programs for PrEP
Work with care team to select individualized HIV treatment regimens
Monitor HIV treatment responses, adverse events, and drug interactions
Identify areas for regimen simplification to improve therapy adherence

*Source:* Human Rights Campaign Foundation.^34^

A central role for pharmacists also exists in the management of hepatitis C therapy among TG individuals, particularly with respect to direct-acting antiviral agents.^89^ TG individuals who share needles, syringes, or vials for hormone injections or illegal drugs, as well as receive unsupervised injections of nonmedical grade soft tissue fillers (i.e., silicone), may be at risk of hepatitis C infection.^90^

The pharmacists can also provide valuable information and pharmacotherapy recommendations to the treatment team regarding the many complex drug–drug interactions between hormonal therapy and HIV medications.^23,91,92^ These complex drug–drug interactions in TG persons can be further complicated when other comorbid diseases (e.g., epilepsy) are also present and HIV medications, antiepileptics, and hormones are coprescribed.^93^ While low doses of ethinyl estradiol alone or combined with progestins in contraceptive pills have a number of interactions with antiretroviral therapy, primarily non-nucleos(t)ide reverse transcriptase inhibitors and protease inhibitors boosted with ritonavir,^91,94^ the extent of these drug–drug interactions with higher estradiol doses typically used in hormone therapy in TG individuals remains unclear. Nevertheless, the perception among many HIV-infected TG women is that antiretroviral therapy may impact the feminizing effects of estrogen therapy. As a result, ∼40% of HIV-infected TG women used feminizing hormone therapies and/or antiretroviral therapy differently than prescribed.^95^ This raises concerns regarding suboptimal adherence to antiretroviral therapy and the possibility of increased risk of developing resistance to antiretroviral therapy, virologic failure, and increased transmission to sexual partners, especially drug-resistant HIV.^96–99^

## Attitudes of Pharmacists Toward TG People

**FIG. 4.**
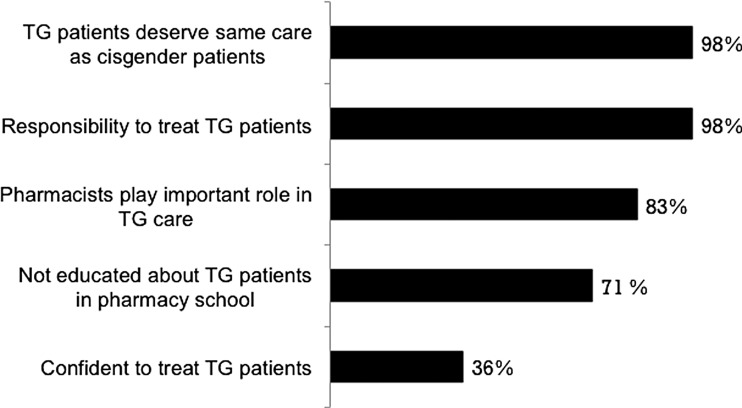
Results of a survey of community pharmacy residents' perceptions of TG health management (*N*=63). Data are extracted from Leach and Layson-Wolf.^27^

Although the attitudes of practicing pharmacists toward TG people have not been extensively investigated, a relatively small survey of community pharmacy residents' perceptions of TG health management provides some valuable insights (Fig. 4).^27^ This cross-sectional, anonymous survey involved a total of 63 pharmacy residents and comprised a 34-question online questionnaire. While most pharmacy residents said that TG patients deserved the same care as cisgender patients and pharmacists play an important role in TG care, only 36% said they felt confident to treat TG patients. This lack of confidence appears to stem from the fact that 71% of respondents received no education about TG patient issues in pharmacy school. This finding is very important because, according to statistics from the National Center for Transgender Equality, National Gay and Lesbian Task Force, almost a third of TG patients, have postponed health care due to a fear of being discriminated against, and half of TG patients who have consulted providers lacked relevant knowledge to address their unique health care needs.^100^

## Helping TG Patients from the Pharmacist's Perspective

Pharmacists displaying professional and nonjudgmental behavior are ideally positioned to address at least some of the health care disparities and medication concerns of TG patients.^10,18,23,24,28^ Pharmacists can help TG patients and their families understand their medications and anticipate and manage side effects, particularly since cross-sex hormone regimens are often administered at doses and durations exceeding recommended regimens.^18,35^ This advice should obviously be tailored to the age of the patient since pediatric/adolescent, adult, and geriatric patients will have differing educational needs. It is important that TG patients understand the risks and benefits as well as typical timelines for physical outcomes of hormone therapy. In addition, parents of TG children should fully understand the pharmacotherapeutic principles of gonadotropin-releasing hormone (GnRH) analogs. Guidelines for hormonal therapies are established and usage before the age of 16 years can be initiated, although there is limited published data before 14 years of age.^15^ The off-label use of GnRH agonists in children with normally timed puberty more than doubled (12–29%) from 2013 to 2016, whereas its use to treat precocious puberty (FDA-approved indication) increased only slightly.^101^ A similar temporal trend showing a dramatic rise in use of histrelin implants (from 0 to 63) among TG children occurred from 2004 to 2016 using data from the Pediatric Health Information System.^102^

Pharmacists can also help coordinate health care, prescription assistance programs, and referrals to providers who are TG friendly. As mentioned earlier, only 11% of TG individuals have all their IDs changed to their preferred name and gender. As a result, many TG people have insurance and identification documents that do not reflect their current name or gender identity. Keep in mind that it is important to know the patient's biologic sex to avoid dispensing, without appropriate counseling, potentially teratogenic agents to TG men of childbearing potential and to use the appropriate creatinine clearance reference values for dosing protocols.

TG peoples' physical, mental, and social health involve respectfully affirming their gender identity.^44^ Pharmacists should therefore use preferred gender identity, name, and pronouns; if they are unsure, it is appropriate to use gender-neutral language or politely ask how they wished to be addressed. Avoid making assumptions about a person's gender identity or sexual orientation from their appearance or how they look or sound. In this regard, pharmacists might want to consider adding a “name- and pronoun-in-use” option, as well as birth sex, and legal name and sex, on intake forms and in pharmacy records to avoid embarrassing misidentifications. Intake forms used in many pharmacies can also be more inclusive by including other gender variations beside the usual male/female binary. TG person's legal versus preferred names may pose challenges for pharmacists and pharmacy electronic prescription computer records that require creative solutions.

Using appropriate language and terminology interacting with TG individuals can be challenging and both a facilitator and barrier to cultural competence.^103^ As noted by Ruben et al., poor cultural competence is a contributing factor to health care disparities, particularly with respect to sexual and gender minority patients.^104^ Wherever possible, pharmacy staff, particularly those at the point of contact with patients, should also be trained in basic cultural awareness of TG people and “de-gender” their language when referring to patients; for example, using they instead of he or she. To further improve the TG-affirming setting, the waiting area should display a nondiscrimination policy visible to patients and include visible TG-related education, publications, and/or posters. In addition, private spaces for consultation with TG individuals are also important to consider.

Access to restrooms by TG persons can be contentious for many cisgender persons. If feasible, pharmacies should consider designating a gender-neutral bathroom or single-stall bathroom as an option. If a unisex restroom is not feasible, consider allowing TG individuals to use whatever bathroom is congruent with their gender identity. Currently, however, a total of 16 states are considering legislation that would restrict access to public multiuser restrooms, locker rooms, and other sex-segregated facilities on the basis of the gender stated on their birth certificates.^105^

## Conclusions

Many TG patients experience challenges in the health care system that leads to disparities in health care and formidable barriers to receiving appropriate and culturally competent care. Pharmacists play an important role in the health care system and provide positive steps to promote the health of their TG patients. By providing unbiased, gender-affirming interactions, improving physical pharmacy environments with policies and procedures that include staff training, pharmacists are uniquely accessible to TG persons. Future research opportunities for pharmacists in the TG health care field abound not only in terms of pharmacotherapeutic regimens but also developing pharmacy-based interventions in health care services (e.g., HIV testing, syringe access, and PrEP) and preventative health interventions (e.g., health fair screenings, vaccinations, blood pressure, cholesterol, smoking cessation).

## Abbreviations Used

DHHS: Department of Health and Human Services
GnRH: gonadotropin-releasing hormone
HIV: human immunodeficiency virus
LGB: lesbian, gay, and bisexual
OSCE: objective structured clinical examination
PEP: postexposure prophylaxis
PrEP: preexposure prophylaxis
STD: sexually transmitted disease
TG: transgender

## References

[1] Technology: will it help or hurt the future of pharmacy practice? American Pharmacists Association. Available at www.pharmacist.com/technology-will-it-help-or-hurt-future-pharmacy-practice (accessed 118, 2018)

[2] Vollmer L. The future of pharmacy. Pharmacy Times. Available at www.pharmacytimes.com/publications/directions-in-pharmacy/2014/december2014/the-future-of-pharmacy (accessed 118, 2018)

[3] Kenreigh CA, Timm Wagner L Pharmacists' role in healthcare still evolving. 2006. Available at www.medscape.com/viewarticle/546717 (accessed 119, 2018)

[4] Marotta R. The evolving role of pharmacy technicians. 2015. Pharmacy Times. Available at www.pharmacytimes.com/technician-news/the-evolving-role-of-pharmacy-technicians (accessed 119, 2018)

[5] Reisner SL, Bradford J, Hopwood R, et al. Comprehensive transgender healthcare: the gender affirming clinical and public health model of Fenway Health. J Urban Health. 2015;92:584–592 25779756 10.1007/s11524-015-9947-2 PMC4456472

[6] Bonner L. Pharmacists can be accessible, trusted providers for transgender patients. Pharmacy Today. Available at www.pharmacytoday.org/article/S1042-0991(16)00356-X/pdf (accessed 119, 2018)

[7] Kaigle A, Sawan-Garcia R, Firek A Approach to the provision of transgender health care in a veteran population. Ment Health Clin. 2018;7:176–180 29955520 10.9740/mhc.2017.07.176 PMC6007524

[8] Redfern JS, Barnes A, Chang J Psychosocial, HIV, and health care management issues impacting transgender individuals. Am J Orthopsychiatry. 2016;86:366–372 27380150 10.1037/ort0000190

[9] Redfern JS, Sinclair B Improving health care encounters and communication with transgender patients. J Comm Healthcare. 2014;7:25–40

[10] Transgender woman says CVS pharmacist refused to fill hormone prescription. *New York Times*, July 20, 2018. Available at www.nytimes.com/2018/07/20/us/cvs-pharmacy-transgender-woman-nyt.html (accessed 725, 2018)

[11] Kibicho J, Owczarzak J A patient-centered pharmacy services model of HIV patient care in community pharmacy settings: a theoretical and empirical framework. AIDS Patient Care STDS. 2012;26:20–28 22149903 10.1089/apc.2011.0212

[12] Koerner PH, Miller RT, Higginbotham S Development of a community residency program with a focus on specialty pharmacy. Am J Health Syst Pharm. 2014;71:2067–2072 25404599 10.2146/ajhp130732

[13] Wilkening GL. The current status of transgender health education in doctor of pharmacy curricula in North America. Ment Health Clin. 2017;7:168–171 29955518 10.9740/mhc.2017.07.168 PMC6007525

[14] Daniel H, Butkus R Lesbian, gay, bisexual, and transgender health disparities: executive summary of a policy position paper from the American College of Physicians. Ann Intern Med. 2015;163:135–137 25961598 10.7326/M14-2482

[15] Hembree WC, Cohen-Kettenis PT, Gooren L, et al. Endocrine treatment of gender-dysphoric/gender-incongruent persons: an Endocrine Society clinical practice guideline. J Clin Endocrinol Metab. 2017;102:3869–3903 28945902 10.1210/jc.2017-01658

[16] Byne W, Bradley SJ, Coleman E, et al. Report of the American Psychiatric Association Task Force on treatment of gender identity disorder. Arch Sex Behav. 2012;41:759–796 22736225 10.1007/s10508-012-9975-x

[17] Okoro O, Odedina F, Smith WT Determining the sufficiency of cultural competence instruction in pharmacy school curriculum. Am J Pharm Educ. 2015;79:5 0 10.5688/ajpe79450 PMC4469016 26089559

[18] Bishop BM. Pharmacotherapy considerations in the management of transgender patients: a brief review. Pharmacotherapy. 2015;35:1130–1139 26684553 10.1002/phar.1668

[19] Daniels CC, Trujillo TN, Scott CM, et al. Navigating the residency application process for lesbian, gay, bisexual, and transgender student pharmacists. Am J Health Syst Pharm. 2018;75:173–176 29326140 10.2146/ajhp170503

[20] Ostroff JL, Ostroff ML, Billings S, et al. Integration of transgender care into a pharmacy therapeutics curriculum. Curr Pharm Teach Learn. 2018;10:463–468 29793708 10.1016/j.cptl.2017.12.016

[21] Owens RE, Wargo KA Transgender students in pharmacy school part 2: how faculty advisors can support their advisees. Curr Pharm Teach Learn. 2017;9:957–961 29233391 10.1016/j.cptl.2017.07.027

[22] Parkhill AL, Mathews JL, Fearing S, et al. A transgender health care panel discussion in a required diversity course. Am J Pharm Educ. 2014;78:8 1 24850943 10.5688/ajpe78481 PMC4028590

[23] Radix AE. Pharmacists' role in provision of transgender healthcare. Am J Health Syst Pharm. 2017;74:103–104 28122749 10.2146/ajhp160939

[24] Cocohoba J. Pharmacists caring for transgender persons. Am J Health Syst Pharm. 2017;74:170–174 27486064 10.2146/ajhp151053

[25] Irving A, Lehault WB Clinical pearls of gender-affirming hormone therapy in transgender patients. Ment Health Clin. 2017;7:164–167 29955517 10.9740/mhc.2017.07.164 PMC6007530

[26] Jacobson AN, Matson KL, Mathews JL, et al. Lesbian, gay, bisexual, and transgender inclusion: survey of campus climate in colleges and schools of pharmacy. Curr Pharm Teach Learn. 2017;9:60–65 29180156 10.1016/j.cptl.2016.08.038

[27] Leach C, Layson-Wolf C Survey of community pharmacy residents' perceptions of transgender health management. J Am Pharm Assoc. 2016;56:44 1.e6–445.e6. 10.1016/j.japh.2016.03.008 27245854

[28] Maxwell E, Salch S, Boliko M, et al. Discrepancies in lesbian, gay, bisexual, and transgender patient care and how pharmacists can support an evolved practice. Am J Pharm Educ. 2017;81:618 1 10.5688/ajpe8176181 PMC5663655 29109564

[29] Newsome C, Colip L, Sharon N, et al. Incorporating a pharmacist into an interprofessional team providing transgender care under a medical home model. Am J Health Syst Pharm. 2017;74:135–139 28122755 10.2146/ajhp160322

[30] Wargo KA, Owens RE Transgender students in pharmacy school, part 1: what academic administrators need to know. Curr Pharm Teach Learn. 2017;9:951–956 29233390 10.1016/j.cptl.2017.07.007

[31] Braun HM, Ramirez D, Zahner GJ, et al. The LGBTQI health forum: an innovative interprofessional initiative to support curriculum reform. Med Educ Online. 2017;22:130641 9 10.1080/10872981.2017.1306419 PMC5419298 28399716

[32] Jann MW, Penzak SR, White A, et al. An elective course in lesbian, gay, bisexual, and transgender (LGBT) health and practice issues. Am J Pharm Educ. 2018;82:49 3 10.5688/ajpe6967 PMC6900814 31831892

[33] Parkhill AL, Gainsburg J, Fearing S, et al. The need for transgender health content in the pharmacy curriculum. Innov Pharm. 2011;11:1–4

[34] Human Rights Campaign Foundation. Providing LGBTQ-inclusive care and services at your pharmacy. A resource guide for pharmacists and pharmacy staff. June 2016. Available at https://issuu.com/humanrightscampaign/docs/lgbtq-pharmacyguide-2016/3 (accessed 118, 2018)

[35] Ross M. 5 Ways pharmacists can help transgender patients. Pharmacy Times. Available at www.pharmacytimes.com/news/5-ways-pharmacists-can-help-transgender-patients?p=2 (accessed 118, 2018)

[36] Flores AR, Herman JL, Gates GJ, Brown TNT How Many Adults Identify as Transgender in the United States? Los Angeles, CA: The Williams Institute. Available at http://williamsinstitute.law.ucla.edu/wp-content/uploads/How-Many-Adults-Identify-as-Transgender-in-the-United-States.pdf (accessed 26, 2018)

[37] James SE, Herman JL, Rankin S, et al. The Report of the 2015 U.S. Transgender Survey. Washington, DC: National Center for Transgender Equality, 2016

[38] Learmonth C, Viloria R, Lambert C, et al. Barriers to insurance coverage for transgender patients. Am J Obstet Gynecol. 2018;219:272–274 29733842 10.1016/j.ajog.2018.04.046

[39] Olson-Kennedy J, Cohen-Kettenis PT, Kreukels BP, et al. Research priorities for gender nonconforming/transgender youth: gender identity development and biopsychosocial outcomes. Curr Opin Endocrinol Diabetes Obes. 2016;23:172–179 26825472 10.1097/MED.0000000000000236 PMC4807860

[40] American Psychological Association. Gender Dysphoria. Diagnostic and Statistical Manual of Mental Disorders 5. 2013. Available at www.scribd.com/document/340209199/APA-DSM-5-Gender-Dysphoria-1 (accessed 118, 2018)

[41] Davy Z. The DSM-5 and the politics of diagnosing transpeople. Arch Sex Behav. 2015;44:1165–1176 26054486 10.1007/s10508-015-0573-6

[42] Drescher J. Transsexualism, gender identity disorder and the DSM. J Gay Lesbian Mental Health. 2010;14:109–122

[43] Drescher J. Gender identity diagnoses: history and controversies. In: Gender Dysphoria and Disorders of Sex Development. Focus on Sexuality Research. (Kreukels B, Steensma T, de Vries A; eds). Boston, MA: Springer, 2014, pp. 137–150

[44] Reisner SL, Radix A, Deutsch MB Integrated and gender-affirming transgender clinical care and research. J Acquir Immune Defic Syndr. 2016;72(Suppl 3):S235–S242 27429189 10.1097/QAI.0000000000001088 PMC4969060

[45] Drescher J. Gender Diagnoses and ICD-11. Psychiatric News. Available at https://psychnews.psychiatryonline.org/doi/full/10.1176/appi.pn.2016.8a15 (accessed 118, 2018)

[46] ICD-11 Beta Version for Mortality and Morbidity Statistics (ICD-11 MMS) 2018 version. Available at https://icd.who.int/browse11/l-m/en (accessed 726, 2018)

[47] Burton N. When Homosexuality Stopped Being a Mental Disorder. Psychology Today. Available at www.psychologytoday.com/blog/hide-and-seek/201509/when-homosexuality-stopped-being-mental-disorder (accessed 118, 2018)

[48] Pandya A. Mental health as an advocacy priority in the lesbian, gay, bisexual, and transgender communities. J Psychiatr Pract. 2014;20:225–227 24847996 10.1097/01.pra.0000450322.06612.a1

[49] Christian R, Mellies AA, Bui AG, et al. Measuring the health of an invisible population: lessons from the Colorado Transgender Health Survey. J Gen Intern Med. 2018;33:1654–1660 29761263 10.1007/s11606-018-4450-6 PMC6153233

[50] American College of Pediatricians. Gender ideology harms children. Available at www.acpeds.org/the-college-speaks/position-statements/gender-ideology-harms-children (accessed 118, 2018)

[51] Green EL, Benner K, Pear R. “Transgender” Could Be Defined Out of Existence Under Trump Administration, The New York Times, October 21, 2018

[52] Savic I, Garcia-Falgueras A, Swaab DF Sexual differentiation of the human brain in relation to gender identity and sexual orientation. Prog Brain Res. 2010;186:41–62 21094885 10.1016/B978-0-444-53630-3.00004-X

[53] Manzouri A, Kosidou K, Savic I Anatomical and functional findings in female-to-male transsexuals: testing a new hypothesis. Cereb Cortex. 2017;27:998–1010 26637450 10.1093/cercor/bhv278

[54] Saraswat A, Weinand JD, Safer JD Evidence supporting the biologic nature of gender identity. Endocr Pract. 2015;21:199–204 25667367 10.4158/EP14351.RA

[55] Theisen JG, Filchak MS, Sundaram V, et al. Understanding the genetic basis of transgender identity. Presented at the Society for Reproductive Investigation's annual meeting in San Diego, March 6–10, 2018

[56] Spizzirri G, Duran FLS, Chaim-Avancini TM, et al. Grey and white matter volumes either in treatment-naive or hormone-treated transgender women: a voxel-based morphometry study. Sci Rep. 2018;8:73 6 29335438 10.1038/s41598-017-17563-z PMC5768734

[57] Guillamon A, Junque C, Gómez-Gil E A review of the status of brain structure research in transsexualism. Arch Sex Behav. 2016;45:1615–1648 27255307 10.1007/s10508-016-0768-5 PMC4987404

[58] Safer JD, Coleman E, Feldman J, et al. Barriers to healthcare for transgender individuals. Curr Opin Endocrinol Diabetes Obes. 2016;23:168–171 26910276 10.1097/MED.0000000000000227 PMC4802845

[59] Horvath KJ, Iantaffi A, Swinburne-Romine R, Bockting W A comparison of mental health, substance use, and sexual risk behaviors between rural and non-rural transgender persons. J Homosex. 2014;61:1117–1130 24380580 10.1080/00918369.2014.872502 PMC4301267

[60] Shires DA, Stroumsa D, Jaffee KD, et al. Primary care providers' willingness to continue gender-affirming hormone therapy for transgender patients. Fam Pract. 2017;35:576–581 10.1093/fampra/cmx119 29236982

[61] Johnston CD, Shearer LS Internal medicine resident attitudes, prior education, comfort, and knowledge regarding delivering comprehensive primary care to transgender patients. Transgend Health. 2017;2:91–95 28861552 10.1089/trgh.2017.0007 PMC5548411

[62] Kaiser Permanente Innovation Consultancy. Understanding healthcare needs of transgender individuals. Available at http://c.ymcdn.com/sites/www.dmi.org/resource/resmgr/design_value_awards/2016DVA-Kaiser.pdf (accessed 118, 2018)

[63] Jaffee KD, Shires DA, Stroumsa D Discrimination and delayed health care among transgender women and men: implications for improving medical education and health care delivery. Med Care. 2016;54:1010–1016 27314263 10.1097/MLR.0000000000000583

[64] Gonzales G, Przedworski J, Henning-Smith C Comparison of health and health risk factors between lesbian, gay, and bisexual adults and heterosexual adults in the United States: results from the National Health Interview Survey. JAMA Intern Med. 2016;176:1344–1351 27367843 10.1001/jamainternmed.2016.3432

[65] Downing JM, Przedworski JM Health of transgender adults in the U.S., 2014–2016. Am J Prev Med. 2018;55:336–344 30031640 10.1016/j.amepre.2018.04.045

[66] HHS announces new conscience and religious freedom division. US Department of Health and Human Services. Conscience and Religious Freedom. Available at www.hhs.gov/about/news/2018/01/18/hhs-ocr-announces-new-conscience-and-religious-freedom-division.html (accessed 719, 2018)

[67] Snelgrove JW, Jasudavisius AM, Rowe BW, et al. “Completely out-at-sea” with “two-gender medicine”: a qualitative analysis of physician-side barriers to providing healthcare for transgender patients. BMC Health Serv Res. 2012;12:11 0 22559234 10.1186/1472-6963-12-110 PMC3464167

[68] Lawrence AA. Factors associated with satisfaction or regret following male-to-female sex reassignment surgery. Arch Sex Behav. 2003;32:299–315 12856892 10.1023/a:1024086814364

[69] Pfafflin F. Regrets after sex reassignment surgery. J Psychol Hum Sex. 1993;5:4:69–85

[70] Landén M, Wålinder J, Hambert G, et al. Factors predictive of regret in sex reassignment. Acta Psychiatr Scand. 1998;97:284–289 9570489 10.1111/j.1600-0447.1998.tb10001.x

[71] Sobh AH, Austin Z, Izham MIM, et al. Application of a systematic approach to evaluating psychometric properties of a cumulative exit-from-degree objective structured clinical examination (OSCE). Curr Pharm Teach Learn. 2017;9:1091–1098 29233377 10.1016/j.cptl.2017.07.011

[72] Bondar A. A new practitioner's perspective on combining interprofessional and cultural competence instruction in pharmacy school curriculum. Am J Pharm Educ. 2015;79:16 0 26889072 10.5688/ajpe7910160 PMC4749908

[73] Chipkin SR, Kim F Ten most important things to know about caring for transgender patients. Am J Med. 2017;130:1238–1245 28716460 10.1016/j.amjmed.2017.06.019

[74] NIH FY 2016–2020 Strategic plan to advance research on the health and well-being of sexual and gender minorities. National Institutes of Health Sexual and Gender Minority Research Coordinating Committee. Available at www.edi.nih.gov/sites/default/files/EDI_Public_files/sgm-strategic-plan.pdf (accessed 118, 2018)

[75] Office of Disease Prevention and Health Promotion. Health People 2020. Lesbian, gay, bisexual, and transgender health. Health.gov. Available at www.healthypeople.gov/2020/topics-objectives/topic/lesbian-gay-bisexual-and-transgender-health-seven (accessed 25, 2018)

[76] Padula WV, Baker K Coverage for gender-affirming care: making health insurance work for transgender Americans. LGBT Health. 2017;4:244–247 28708447 10.1089/lgbt.2016.0099

[77] Nahata L, Chelvakumar G, Leibowitz S Gender-affirming pharmacological interventions for youth with gender dysphoria: when treatment guidelines are not enough. Ann Pharmacother. 2017;51:1023–1032 28660776 10.1177/1060028017718845

[78] Roberts TK, Kraft CS, French D, et al. Interpreting laboratory results in transgender patients on hormone therapy. Am J Med. 2014;127:159–162 24332725 10.1016/j.amjmed.2013.10.009

[79] Schiff GD, Hasan O, Kim S, et al. Diagnostic error in medicine: analysis of 583 physician-reported errors. Arch Intern Med. 2009;169:1881–1887 19901140 10.1001/archinternmed.2009.333

[80] Baral SD, Poteat T, Stromdahl S, et al. Worldwide burden of HIV in transgender women: a systematic review and meta-analysis. Lancet Infect Dis. 2013;13:214–222 23260128 10.1016/S1473-3099(12)70315-8

[81] Poteat T, Scheim A, Xavier J, et al. Global epidemiology of HIV infection and related syndemics affecting transgender people. J Acquir Immune Defic Syndr. 2016;72(Suppl 3):S210–S219 27429185 10.1097/QAI.0000000000001087 PMC4969059

[82] Reback CJ, Fletcher JB HIV prevalence, substance use, and sexual risk behaviors among transgender women recruited through outreach. AIDS Behav. 2014;18:1359–1367 24287786 10.1007/s10461-013-0657-z PMC4209944

[83] Transgender people HI V and AIDS avert Global information and education on HIV and AIDS. Available at www.avert.org/professionals/hiv-social-issues/key-affected-populations/transgender (accessed 120, 2018)

[84] Transgender people and HIV. World Health Organization, July 2015. Available at http://apps.who.int/iris/bitstream/10665/179517/1/WHO_HIV_2015.17_eng.pdf?ua=1&ua=1 (accessed 120, 2018)

[85] Siskind RL, Andrasik M, Karuna ST, et al. Engaging transgender people in NIH-Funded HIV/AIDS clinical trials research. J Acquir Immune Defic Syndr. 2016;72(Suppl 3):S243–S247 27429190 10.1097/QAI.0000000000001085 PMC4969066

[86] Poteat TC, Keatley J, Wilcher R, et al. Evidence for action: a call for the global HIV response to address the needs of transgender populations. J Int AIDS Soc. 2016;19:2119 3 10.7448/IAS.19.3.21193 PMC4949318 27431476

[87] Neumann MS, Finlayson TJ, Pitts NL, et al. Comprehensive HIV prevention for transgender persons. Am J Public Health. 2017;107:207–212 27997228 10.2105/AJPH.2016.303509 PMC5227924

[88] Meyerson BE, Emetu RE, Sanders SA, et al. Preferences of gay and bisexual men for pharmacy-based HIV testing and over-the-counter HIV tests. LGBT Health. 2014;1:225–228 26789716 10.1089/lgbt.2014.0010

[89] Sebhatu P, Martin MT Genotype 1 hepatitis C virus and the pharmacist's role in treatment. Am J Health Syst Pharm. 2016;73:764–774 27126832 10.2146/ajhp150704

[90] Poteate T. Guidelines for the primary and gender-affirming care of transgender and gender nonbinary people: transgender health and hepatitis C. Available at http://transhealth.ucsf.edu/trans?page=guidelines-hepatitis-c (accessed 117, 2019)

[91] Tittle V, Bull L, Boffito M, et al. Pharmacokinetic and pharmacodynamic drug interactions between antiretrovirals and oral contraceptives. Clin Pharmacokinet. 2015;54:23–34 25331712 10.1007/s40262-014-0204-8

[92] Landolt NK, Phanuphak N, Ubolyam S, et al. Significant decrease of ethinylestradiol with nevirapine, and of etonogestrel with efavirenz in HIV-positive women. J Acquir Immune Defic Syndr. 2014;66:e50–e52 24608892 10.1097/QAI.0000000000000134

[93] Johnson EL, Kaplan PW Caring for transgender patients with epilepsy. Epilepsia. 2017;58:1667–1672 28771690 10.1111/epi.13864

[94] Robinson JA, Jamshidi R, Burke AE Contraception for the HIV-positive woman: a review of interactions between hormonal contraception and antiretroviral therapy. Infect Dis Obstet Gynecol. 2012;2012:89016 0 10.1155/2012/890160 PMC3426212 22927715

[95] Braun HM, Candelario J, Hanlon CL, et al. Transgender women living with HIV frequently take antiretroviral therapy and/or feminizing hormone therapy differently than prescribed due to drug-drug interaction concerns. LGBT Health. 2017;4:371–375 28876170 10.1089/lgbt.2017.0057 PMC5661861

[96] Sethi AK, Celentano DD, Gange SJ, et al. Association between adherence to antiretroviral therapy and human immunodeficiency virus drug resistance. Clin Infect Dis. 2003;37:1112–1118 14523777 10.1086/378301

[97] Bezabhe WM, Chalmers L, Bereznicki LR, et al. Adherence to antiretroviral therapy and virologic failure: a meta-analysis. Medicine (Baltimore). 2016;95:e336 1 10.1097/MD.0000000000003361 PMC4839839 27082595

[98] Kozal MJ, Amico KR, Chiarella J, et al. Antiretroviral resistance and high-risk transmission behavior among HIV-positive patients in clinical care. AIDS. 2004;18:2185–2189 15577652 10.1097/00002030-200411050-00011

[99] Soeters HM, Napravnik S, Zakharova OM, et al. Opportunities for sexual transmission of antiretroviral drug resistance among HIV-infected patients in care. AIDS. 2013;27:2873–2881 23921618 10.1097/01.aids.0000433240.78739.30 PMC3916948

[100] National Center for Transgender Equality. National Gay and Lesbian Task Force. Injustice at every turn: a report of the national transgender discrimination survey. Available at www.thetaskforce.org/static_html/downloads/reports/reports/ntds_full.pdf (accessed 117, 2018)

[101] Lopez CM, Solomon D, Boulware SD, et al. Trends in the “off-label” use of GnRH agonists among pediatric patients in the United States. Clin Pediatr (Phila). 2018;57:1432–1435 30003804 10.1177/0009922818787260

[102] Lopez CM, Solomon D, Boulware SD, et al. Trends in the use of puberty blockers among transgender children in the United States. J Pediatr Endocrinol Metab. 2018;31:665–670 29715194 10.1515/jpem-2018-0048

[103] Rossi AL, Lopez EJ Contextualizing competence: language and LGBT-based competency in health care. J Homosex. 2017;64:1330–1349 28467155 10.1080/00918369.2017.1321361

[104] Ruben MA, Shipherd JC, Topor D, et al. Advancing LGBT health care policies and clinical care within a large academic health care system: a case study. J Homosex. 2017;64:1411–1431 28459380 10.1080/00918369.2017.1321386

[105] “Bathroom Bill” legislative tracking. National Conference of State Legislatures. Available at www.ncsl.org/research/education/-bathroom-bill-legislative-tracking635951130.aspx (accessed 118, 2018)

